# Timing of Antiretroviral Therapy Initiation after a First AIDS-Defining Event: Temporal Changes in Clinical Attitudes in the ICONA Cohort

**DOI:** 10.1371/journal.pone.0089861

**Published:** 2014-02-27

**Authors:** Antonella Cingolani, Alessandro Cozzi-Lepri, Adriana Ammassari, Cristina Mussini, Maria Alessandra Ursitti, Pietro Caramello, Gioacchino Angarano, Paolo Bonfanti, Andrea De Luca, Maria Stella Mura, Enrico Girardi, Andrea Antinori, Antonela D'Arminio Monforte, for Icona Foundation Study group

**Affiliations:** 1 Department of Public Health, Institute of Infectious Diseases, Catholic University, Roma, Italy; 2 Research Department of Infection & Population Health, University College London, London, United Kingdom; 3 Clinical Department, National Institute for Infectious Diseases “L. Spallanzani,” Roma, Italy; 4 Institute of Infectious Diseases, University of Modena and Reggio Emilia, Modena, Italy; 5 Department of Infectious Diseases, S. Maria Nuova IRCCS Hospital, Reggio Emilia, Italy; 6 Infectious and Tropical Diseases Unit I, Department of Infectious Diseases, Amedeo di Savoia Hospital, Torino, Italy; 7 Department of Infectious Diseases, University of Bari, Bari, Italy; 8 Unit of Infectious Diseases, A. Manzoni Hospital, Lecco, Italy; 9 Department of Internal and Specialty Medicine, University Infectious Diseases Unit, Azienda Ospedaliera Universitaria Senese, Siena, Italy; 10 Department of Infectious Diseases, University of Sassari, Sassari, Italy; 11 Department of Epidemiology, National Institute for Infectious Diseases “L. Spallanzani,” Roma, Italy; 12 Department of Medicine, Surgery and Dentistry University of Milan Clinic of Infectious Diseases, “San Paolo” Hospital, Milan, Italy; Hopital Bichat Claude Bernard, France

## Abstract

**Background:**

Time of starting antiretroviral therapy (ART) after diagnosis of specific AIDS-defining event (ADE) is a crucial aspect. Objectives of this study were to evaluate if in patients diagnosed with ADE the time to ART initiation may vary according to year of diagnosis and type of ADE.

**Methods:**

All HIV+ persons diagnosed with an ADE over the 6 months prior to or after enrolment in the Icona Foundation study cohort and while ART-naive were grouped according to type of diagnosis: Those with ADE requiring medications interacting with ART [group A], those with ADE treatable only with ART [B] and other ADE [C]. Survival analysis by Kaplan-Meier was used to estimate the percentage of people starting ART, overall and after stratification for calendar period and ADE group. Multivariable Cox regression model was used to investigate association between calendar year of specific ADE and time to ART initiation.

**Results:**

720 persons with first ADE were observed over 1996–2013 (group A, n = 171; B, n = 115; C, n = 434). By 30 days from diagnosis, 27% (95% CI: 22–32) of those diagnosed in 1996–2000 had started ART vs. 32% (95% CI: 24–40) in 2001–2008 and 43% (95% CI: 33–47) after 2008 (log-rank p = 0.001). The proportion of patients starting ART by 30 days was 13% (95% CI 7–19), 40% (95% CI: 30–50) and 38% (95% CI 33–43) in ADE groups A, B and C (log-rank p = 0.0001). After adjustment for potential confounders, people diagnosed after 2008 remained at increased probability of starting ART more promptly than those diagnosed in 1996–1999 (AHR 1.72 (95% CI 1.16–2.56).

**Conclusions:**

In our “real-life” setting, the time from ADE to ART initiation was significantly shorter in people diagnosed in more recent years, although perhaps less prompt than expected.

## Introduction

A large proportion of HIV-infected people still present for care with low CD4 cell count or with an AIDS-defining event (ADE) at first diagnosis of HIV infection [1]. This represents a population with higher probability of clinical progression and death [2] and lower chances of immunological recovery [3]. The optimal timing of starting ART for people presenting with ADE has been debated for a long time. The results of the AIDS Clinical Trials Group (ACTG) protocol 5164 showed that starting antiretroviral therapy within the first 30 days after a diagnosis of opportunistic infections (OI) other than tuberculosis reduces AIDS progression and death by 50% [4] compared to delayed initiation. Moreover, a subsequent analysis of the same trial demonstrated that early initiation is also a cost-effective approach [5]. Following the presentation of ACTG findings, a number of national and international guidelines have been modified accordingly. In particular, currently the Italian guidelines state that people with different ADE (except for meningeal tuberculosis and criptococcosis) should start ART within 30 days from ADE diagnosis [6]–[8]. Nevertheless, the extent to which clinicians in ‘real life’ strictly follow these guidelines remains unexplored. Preliminary results on people diagnosed with *Pneumocystis jiroveci* pneumonia (Pcp) demonstrated that it may be feasible to treat these patients very early [9]. Nevertheless, the risk of overlapping toxicities, as well as pharmacokinetics/pharmacodinamics interactions between antiretrovirals and specific treatment of OI, and the high pill burden with subsequent risk of poor adherence may all represent factors limiting the strict implementation of these new recommendations [10]–[13]. In order to evaluate the possible impact of changes in Italian guidelines following the dissemination of the results of trials such as ACTG 5164, we analysed temporal changes of the time from a first diagnosis of ADE to the time of starting antiretroviral treatement (ART) in patients of the Icona Foundation Study cohort who were diagnosed with AIDS when ART-naive.

## Methods

### Study population

**Figure 1:**
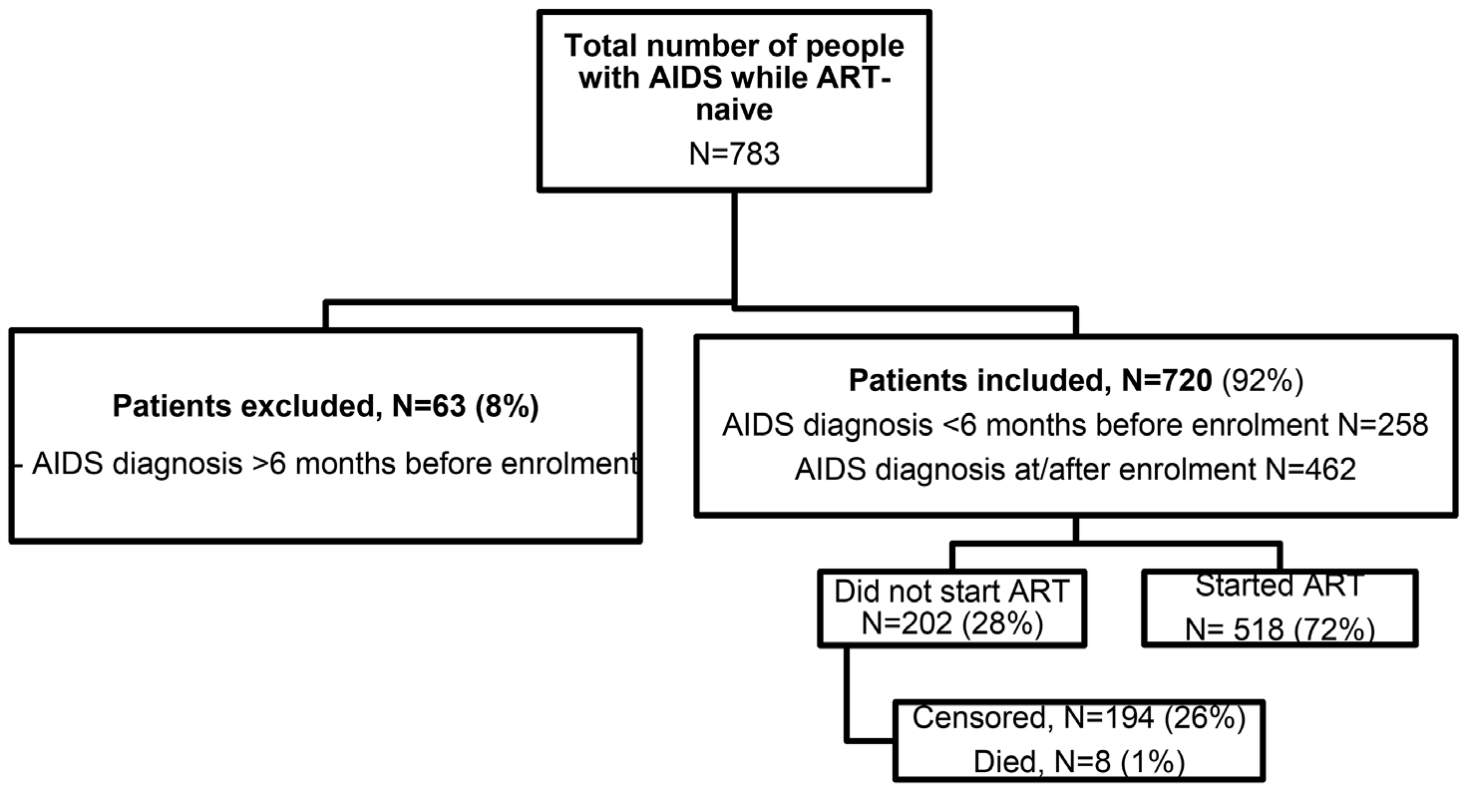
Flow diagram showing the original population of people diagnosed with AIDS while ART-naive, patients definitively considered for the analysis, and reasons for exclusion.

All HIV-1 infected patients of the ICONA Foundation Study who were diagnosed with AIDS while ART-naïve regardless of time of enrolment in the cohort and of their CD4 cell count were considered for this analysis (eligible patients). ICONA Foundation Study is an observational cohort of HIV-infected individuals who are antiretroviral naïve at the time of enrolment [14]. This cohort was set up in January, 1997 and to date consists of more than 10,000 patients from 50 infectious disease units in Italy. Initiation and discontinuation dates of each antiretroviral drug, HIV-viral load and CD4 cell count at each clinical visit (every 4–6 months on an average) were recorded for each enrolled patient. AIDS-defining diseases are recorded in the database at the date that this diagnosis is confirmed or presumptive according to Centers for Disease Control and Prevention (CDC) criteria [15]. Of the eligible patients, only individuals who were diagnosed over the 6 months prior to enrolment in the cohort or those diagnosed under prospective follow-up in the cohort and while still ART-naïve were included in this analysis (Flow Chart, Figure 1).

### Ethics statements

All individuals signed an informed consent prior to enrollment and the study was approved by the Ethics Committee of each participating institution that are listed in the Aknowledgments.

### Statistical analysis

Patients included were classified in 3 groups according to the type of AIDS diagnosis observed using a ‘in house’ algorithm for classification. Specifically, the groups were defined ‘a priori’ on the basis of the expected probability of prompt initiation of ART: Group A) OIs treated with drugs that may have interaction with ART such as for example rifamicins and antineoplastic drugs with both NNRTI and PIs: tuberculosis (TB), atypical mycobacteriosis, non-Hodgkin lymphoma (NHL); Group B) ADE treatable mainly by ART: progressive multifocal leucoencephalopathy (PML), Kaposi's sarcoma (KS), wasting syndrome, cryptosporidiasis,; and Group C) all other ADEs (i.e. Pcp, Cytomegalovirus retinitis, toxoplasmic encephalitis, cervical cancer, criptococcosis, esophageal candidiasis). The expected timing of starting ART was the slowest for group A diseases, prompt for group B and intermediate for group C.

Notwithstanding specific treatment for some conditions included in group C may potentially interact with ART but these interactions have not been demonstrated at a pharmacokinetic level.

The inclusion of Kaposi sarcoma in group B might be arbitrary, nevertheless, even though international treatment guidelines recommend the use of polichemotherapy (PCT) together with ART for people with moderate to severe disease (≥T1), in clinical practice, starting ART represents the most rapid curative intervention for any patient presenting with KS.

Further, different indications for timing of starting ART for NHL and TB (both included in group A) are reported in updated guidelines and could have influenced the decision of clinicians; however, considering that the calendar periods of observation are very different in the studied population, we decided to use the potential risk of drug-drug interaction as a more homogeneous “a priori” criterion used by clinicians to decide either to start ART or not.

In case of multiple diagnoses, clinicians are asked to rank the illness in order of disease severity, and the most severe condition was used for classification in the analysis (data not shown).

ART was defined as a regimen of ≥1 drug belonging to one of the 3 major historical drug classes (NRTI, NNRTI and PI).

Calendar periods of AIDS diagnosis were grouped as 1996–2000, 2001–2008, and 2009+, the earliest period reflecting the years of old generation ART regimens typically with a heavy pill burden and lower tolerability, the latter period reflecting the years after the introduction of modern regimens and following the first presentation of the results of the ACTG 5164 trial at an International Conference [16].

Standard survival analysis by Kaplan-Meier was used to estimate the cumulative percentage of people starting ART from the date of AIDS diagnosis (overall and after stratification for both calendar periods and type of ADEs).

All KM plots have been truncated at 30 days. The threshold of 30 days were based on current treatment guidelines as well as assumptions. Thirty days is the maximum length of time whitin which is recommended to start ART after a diagnosis of all opportunistic conditions, except for tuberculosis and criptococcosis.

Multivariable Cox regression model was used to investigate the association between calendar periods of diagnosis and type of ADE with time to ART initiation after controlling for age, gender, nation of birth, HIV transmission route, hepatitis co-infection status, reason for enrolment in the cohort, CD4 count at diagnosis and number of concomitant ADE (single vs. multiple). All demographics as well as laboratory markers that were associated in univariable analysis with a p-value = 0.15 were included in the multivariable model with the exception of CD8 and white blood cell counts which were collinear with CD4 counts.

## Results

### Baseline patients characteristics

A total of 720 individuals diagnosed with AIDS over the 6 months prior to enrolment in the cohort or under prospective follow-up while still ART-naïve were included in this analysis. In included patients, the mean time between HIV and AIDS diagnosis was 32 (SD = 56) months and between enrolment and AIDS diagnosis was 3 (SD = 15) months. Selected characteristics of the study population overall and according to periods of AIDS diagnosis are shown in Table 1. People who were diagnosed with AIDS in 2009 or after were less likely to be Italian born (p<0.001), intravenous drug users (p<0.001) and co-infected with HCV (p<0.001) than those diagnosed in previous years (Table 1).

**Table 1:** Characteristics of patients according to time period of AIDS diagnosis.

Characteristics at AIDS diagnosis	1996–2000	2001–2008	2009+	p-value*	Total
	N = 332	N = 172	N = 216		N = 720
***Age, years***					
Median (IQR)	37 (32, 43)	40 (34, 47)	39 (34, 47)	<.001	38 (33, 45)
***Gender, n(%)***					
Female	86 (25.9%)	58 (33.7%)	58 (26.9%)	0.161	202 (28.1%)
***Nation of birth, n(%)***					
Italian	296 (89.2%)	138 (80.2%)	142 (65.7%)	<.001	576 (80.0%)
***Mode of HIV Transmission, n(%)***				<.001	
IDU	123 (37.0%)	26 (15.1%)	18 (8.3%)		167 (23.2%)
Homosexual contacts	72 (21.7%)	38 (22.1%)	52 (24.1%)		162 (22.5%)
Heterosexual contacts	112 (33.7%)	89 (51.7%)	123 (56.9%)		324 (45.0%)
Other/Unknown	25 (7.5%)	19 (11.0%)	23 (10.6%)		67 (9.3%)
***Calendar year of AIDS diagnosis***					
Median (range)	1998 (1996, 2000)	2004 (2001, 2008)	2011 (2009, 2013)	<.001	2002 (1996, 2013)
***HBsAg, n(%)***				<.001	
Negative	283 (85.2%)	147 (85.5%)	154 (71.3%)		584 (81.1%)
Positive	6 (1.8%)	4 (2.3%)	2 (0.9%)		12 (1.7%)
Not tested	43 (13.0%)	21 (12.2%)	60 (27.8%)		124 (17.2%)
***HCVAb, n(%)***				<.001	
Negative	164 (49.4%)	112 (65.1%)	136 (63.0%)		412 (57.2%)
Positive	136 (41.0%)	37 (21.5%)	21 (9.7%)		194 (26.9%)
Not tested	32 (9.6%)	23 (13.4%)	59 (27.3%)		114 (15.8%)
***CD4 count, cells/mmc***					
Median (IQR)	60 (24, 150)	57 (24, 221)	51 (17, 165)	0.689	56 (22, 158)
***Viral load, log10 copies/mL***					
Median (IQR)	5.31 (4.80, 5.77)	5.14 (4.25, 5.56)	5.30 (4.67, 5.71)	0.065	5.27 (4.66, 5.70)
***CD8 count, cells/mmc***					
Median (IQR)	494 (285, 791)	512 (311, 917)	580 (272, 1052)	0.603	526 (292, 889)
***CD4/CD8 ratio, cells/mmc***					
Median (IQR)	0.12 (0.06, 0.22)	0.15 (0.07, 0.34)	0.14 (0.05, 0.30)	0.533	0.13 (0.06, 0.27)
***ADE group, n(%)***				0.676	
Group A	86 (25.9%)	35 (20.3%)	50 (23.1%)		171 (23.8%)
Group B	49 (14.8%)	29 (16.9%)	37 (17.1%)		115 (16.0%)
Group C	197 (59.3%)	108 (62.8%)	129 (59.7%)		434 (60.3%)
***Haemglobin, g/dL***					
Median (IQR)	11 (10, 13)	11 (10, 13)	12 (10, 13)	0.682	11 (10, 13)
***White Blood Cell, cells/mmc***					
Median (IQR)	4000 (2980, 6200)	4700 (3500, 6200)	4570 (3155, 6665)	0.161	4390 (3100, 6230)
***ALT, IU/L***					
Median (IQR)	31 (22, 51)	36 (21, 52)	34 (21, 58)	0.472	32 (21, 53)
***AST, IU/L***					
Median (IQR)	35 (23, 56)	33 (24, 50)	36 (25, 57)	0.845	35 (24, 56)
***eGFR, 100 mls/min/1.73 m^2^***					
Median (IQR)	88.94 (70.60, 92.93)	107.7 (87.27, 123.5)	105.3 (91.11, 128.7)	0.037	104.4 (87.27, 124.5)
***Reason for enrolment, n(%)***				<.001	
No indications for ART	23 (6.9%)	21 (12.2%)	5 (2.4%)		49 (6.9%)
Patients decision	65 (19.6%)	7 (4.1%)	10 (4.9%)		82 (11.5%)
Contraindications	4 (1.2%)	1 (0.6%)	2 (1.0%)		7 (1.0%)
Recent HIV diagnosis	167 (50.3%)	123 (71.5%)	163 (79.1%)		453 (63.8%)
Recent access to care	60 (18.1%)	16 (9.3%)	22 (10.7%)		98 (13.8%)
Physician decision	13 (3.9%)	3 (1.7%)	0 (0.0%)		16 (2.3%)
Unknown	0 (0.0%)	1 (0.6%)	4 (1.9%)		5 (0.7%)
***Time from enrolment to ADE, days***					
Median (range)	0 (0, 1243)	0 (0, 3685)	0 (0, 4358)	<.001	0 (0, 4358)
***Calendar year of HIV diagnosis***					
Median (IQR)	1997 (1991, 1998)	2003 (2000, 2005)	2011 (2010, 2012)	<.001	2000 (1997, 2009)

^*^Chi-square or Wilcoxon test as appropriate.

Of the 720 individuals studied, 171 (24%) had a main diagnosis of group A, 115 (16%) of group B and the remaining 434 (60%) of group C. 120 patients (17%) were diagnosed with multiple ADE. Patients in group B were older (p<0.001), more likely to be of Italian origin (p<0.001), whereas patients in Group A had lower CD4 cell count at the time of AIDS diagnosis (both p<0.001) than patients with OIs classified in other groups (Table S1).

Overall, considering only the major diagnosis for each individual in those with multiple ADE according to the criterion of severity described in the Methods, the prevalence of specific opportunistic conditions were 32% for Pneumocystis jrovecii pneumonia (Pcp, n = 231), 16% for tuberculosis (TB, n = 115), 12% for esophageal candidiasis (n = 84), 5.4% for CMV disease (n = 39), 7% for Kaposi sarcoma (n = 50), 6.3% for toxoplasma encephalitis (n = 45), 7% for wasting syndrome (n = 23); 5.4% for non Hodgkin Lymphoma (NHL, n = 39), all other conditions were observed at a frequency less than 3%. We then assessed trends over time for specific diagnosis with at least 30 patients being diagnosed. A trend for decreasing prevalence was observed for esophageal candidiasis (46%, 19%, 34% respectively, in the 3 periods), Pcp (45%, 25%, 29%) and TB (54%, 18%, 26%). In contrast, a stable prevalence was seen for CMV (23%, 36% and 41%) and KS (32%, 24% and 44%) and for NHL (31%, 31%, 38%).

### Analysis of time to starting cART according to different periods and groups of ADEs

Overall, 518 people (72%) started ART over follow-up after the AIDS diagnosis; by 30 days from the AIDS diagnosis 33% started ART (95% CI: 29–37).

Out of 518 persons starting ART, 94 persons (19%) started a regimen containing a 2NRTI+NNRTI, 167 (32%) a 2NRTI+PI without ritonavir boosting, 170 (33%) a 2NRTI+PI/r and 87 (17%) other types of regimen (these include 61 people starting less than 3 drugs-53 with 2NRTI alone).

**Figure 2:**
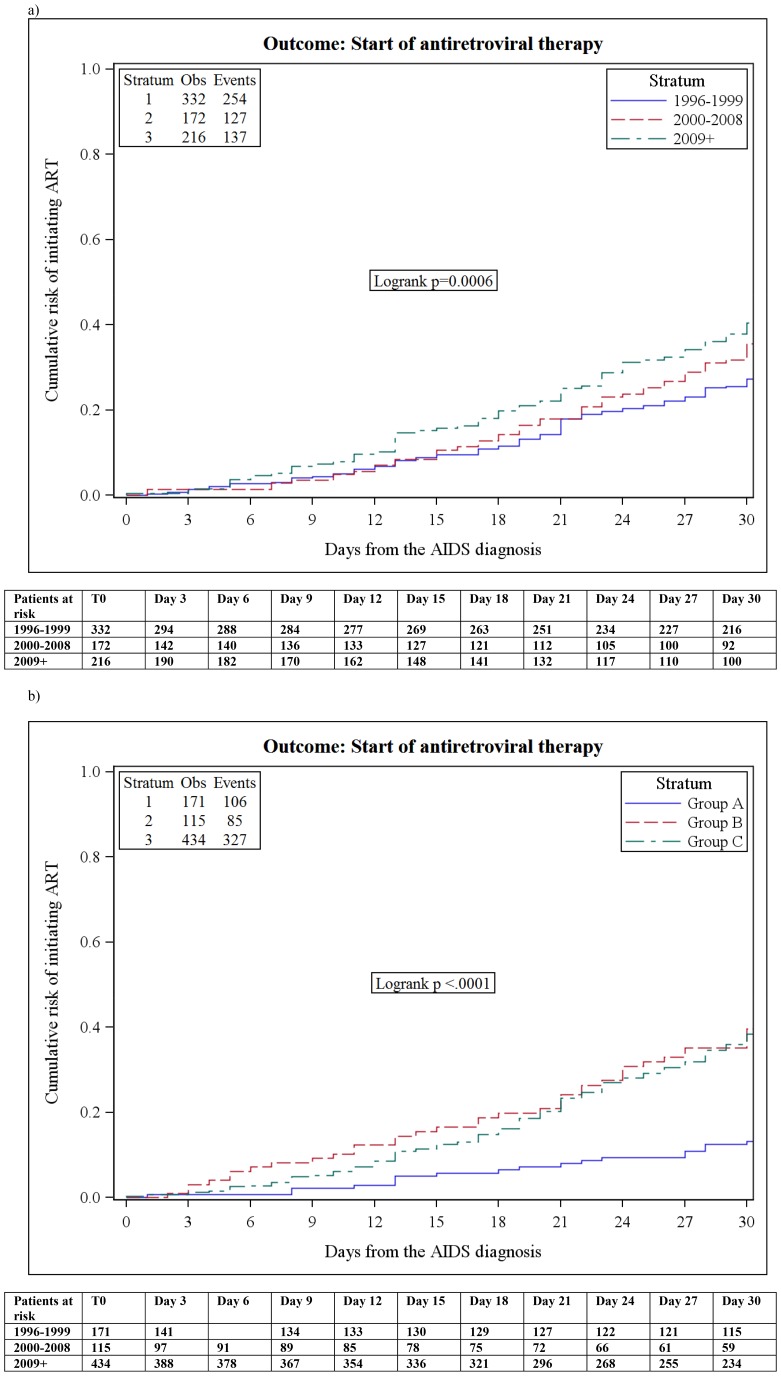
Kaplan-Meier estimates of the cumulative proportion of patients starting ART by 30 days from ADE diagnosis, according to period of starting ART (a) and group of ADEs (b).

Figure 2 shows the Kaplan Meier estimates of the cumulative probability of starting ART, according to the calendar period of starting ART (Figure 2a) and to group of ADEs (Figure 2b). The Kaplan Meier estimate of the median time to ART initiation was 58 days [95% CI: 48–70] for patients diagnosed in the period 1996–1999, 45 days [95%CI: 34–51] for those diagnosed during 2000–2008, and 38 days [95%CI: 33–48] for those with a diagnosis of AIDS in/after 2009. The proportions of people starting ART by 30 days in these same groups were 27% (95% CI:22–32) of those diagnosed in 1996–2000, 32% (95% CI:24–40) of those in 2001–2008 and 43% (95% CI:33–47) of those in/after 2009 (log-rank p = 0.001, Figure 2a).

Patients in group A started ART after a median time of 116 days [95%CI 82–148], those in group B after a median of 35 days [95%CI 30–49] and those in group C after 38 days [95%CI 35–41, log-rank p = 0.0002, Figure 2b). The proportion of patients starting ART by 30 days was 13% (95% CI 7–19), 40% (95% CI: 30–50) and 38% (95% CI 33–43) in ADE groups A, B and C, respectively. After concomitantly stratifying for both these variables, people belonging to group B diagnosed in or after 2009 had the highest overall probability of starting ART by 30 days (51%; 95%CI: 33–69) (Figure S1, data not shown). Of note, the Kaplan-Meier estimate of the median time to starting ART in the whole Icona cohort (11,303 individuals enrolled) is much longer, at 157 months (95% CI:139–173).

After adjusting for a number of potential confounding factors listed in the Methods, people diagnosed in 2009 or after, remained at significantly increased probability of starting ART more promptly than those who were diagnosed in 1996–1999: adjusted HR (AHR) 1.72 (95% CI 1.16–2.56; p = 0.007). The strongest calendar year effect seemed to have occurred for diagnoses belonging to group A (AHR 3.61; 95%CI 1.48–8.77; p = 0.005) (Table 2a).

**Table 2:** Relative Hazards (RH) of starting ART from fitting a Cox regression analysis, according to calendar periods and stratified by type of ADE.

	Crude and adjusted relative hazards of ART initiation			
	Crude RH (95% CI)	p-value	Adjusted* RH (95% CI)	p-value
***All patients***				
***Calendar Period***				
1996–1999	1.00		1.00	
2000–2008	1.19 (0.96, 1.48)	0.104	0.92 (0.67, 1.26)	0.610
2009+	1.51 (1.22, 1.86)	<.001	1.72 (1.16, 2.56)	0.007
***Group A*** a				
***Calendar Period***				
1996–1999	1.00		1.00	
2000–2008	1.09 (0.66, 1.81)	0.726	1.24 (0.59, 2.61)	0.578
2009+	2.38 (1.49, 3.81)	<.001	3.61 (1.48, 8.77)	0.005
***Group B*** b				
***Calendar Period***				
1996–1999	1.00		1.00	
2000–2008	0.95 (0.55, 1.65)	0.851	0.39 (0.13, 1.16)	0.090
2009+	1.63 (0.98, 2.69)	0.058	1.23 (0.37, 4.02)	0.736
***Group C*** c				
***Calendar Period***				
1996–1999	1.00		1.00	
2000–2008	1.21 (0.93, 1.57)	0.156	0.89 (0.58, 1.39)	0.619
2009+	1.26 (0.96, 1.66)	0.095	1.32 (0.74, 2.37)	0.342

^*^adjusted for age, gender, nation of birth, mode of HIV transmission,hepatitis co-infection status, type of AIDS diagnosis-all patients model only-,reason for enrolment in Icona, CD4 count and viral load at diagnosis, number of concomitant AIDS diagnoses

a mycobacteriosis, tubercolosis, Non-Hodgkin lymphomas

b isosporidiosis, criptosporidiosis, PML, Kaposi sarcoma andAIDS dementia complex

c all other ADE

Moreover, after controlling for potential confounders, time to ART initiation was shorter in people belonging to group B (ARH 1.63, 95% CI:1.05–2.53; p = 0.02) compared to patients in group A. The difference in time to start of ART between groups B and A was consistent across calendar periods. (p-value for interaction p = 0.19, Table 3).

**Table 3:** Relative hazards (RH) of starting ART according with groups of ADEs from fitting a Cox regression analysis and stratified by calendar period.

	Crude and adjusted relative hazards of ART initiation			
	Crude RH (95% CI)	p-value	Adjusted* RH (95% CI)	p-value
***All patients***				
***Type of ADE***				
Group A^a^	1.00		1.00	
Group B^b^	2.18 (1.63, 2.90)	<.001	1.63 (1.05, 2.53)	0.028
Group C^c^	2.04 (1.64, 2.55)	<.001	1.01 (0.71, 1.42)	0.962
***1996*** **–** ***1999***				
***Type of ADE***				
Group A^a^	1.00		1.00	
Group B^b^	2.30 (1.52, 3.48)	<.001	1.91 (1.04, 3.52)	0.037
Group C^c^	2.18 (1.60, 2.96)	<.001	1.22 (0.78, 1.91)	0.392
***2000*** **–** ***2008***				
***Type of ADE***				
Group A^a^	1.00		1.00	
Group B^b^	2.03 (1.08, 3.84)	0.029	1.55 (0.55, 4.40)	0.408
Group C^c^	2.42 (1.48, 3.97)	<.001	1.00 (0.40, 2.51)	0.998
***2009+***				
***Type of ADE***				
Group A^a^	1.00		1.00	
Group B^b^	1.99 (1.18, 3.36)	0.010	5.37 (1.08, 26.81)	0.040
Group C^c^	1.55 (1.01, 2.38)	0.047	1.95 (0.52, 7.39)	0.325

^*^adjusted for age, gender, nation of birth, mode of HIV transmission,hepatitis co-infection status, calendar period-all patients model only-,reason for enrolment in Icona, CD4 count and viral load at diagnosis, number of concomitantAIDS diagnoses

a mycobacteriosis, tubercolosis, Non-Hodgkin lymphomas

b isosporidiosis, criptosporidiosis, PML, Kaposi sarcoma andAIDS dementia complex

c all other ADE

### Analysis of other predictors of starting ART

We identified other factors that were associated with the time of ART initiation independent of calendar year and type of AIDS diagnosis from fitting a Cox regression analysis (Table 4). People born in Italy (vs. non Italian-born ARH = 1.83, 95% CI:1.21–2.78) those with lower CD4 cell count at the time of their ADE diagnosis (per 100 cells/mm3 lower AHR = 1.22 [95%CI 1.11–1.34] and people who were enrolled in the cohort because recently discovered to be HIV-infected (vs. those with no indications for starting ART at enrolment, ARH = 1.86, 95% CI:1.17–2.93) were significantly more likely to start ART. Interestingly, viral load at the time of AIDS diagnosis was not independently associated with the probability of starting ART.

**Table 4:** Other factors associated with the chance of starting ART - Relative hazards (RH) from fitting a Cox regression.

	Crude and adjusted relative hazards of ART initiation			
	Crude RH (95% CI)	p-value	Adjusted^*^ RH (95% CI)	p-value
***Age***				
per 10 year older	1.20 (1.09, 1.31)	<.001	0.97 (0.84, 1.12)	0.652
***Gender, n(%)***				
Male	1.00		1.00	
Female	0.86 (0.71, 1.04)	0.122	1.14 (0.83, 1.55)	0.422
***Nation of birth, n(%)***				
Non Italian	1.00		1.00	
Italian	1.50 (1.19, 1.90)	<.001	1.83 (1.21, 2.78)	0.004
***Mode of HIV Transmission, n(%)***				
IDU	1.00		1.00	
Homosexual contacts	1.36 (1.05, 1.75)	0.018	1.11 (0.69, 1.79)	0.664
Heterosexual contacts	1.49 (1.19, 1.87)	<.001	1.07 (0.65, 1.76)	0.797
Other/Unknown	1.47 (1.04, 2.07)	0.029	1.02 (0.55, 1.89)	0.956
***Calendar Period***				
1996–1999	1.00		1.00	
2000–2008	1.19 (0.96, 1.48)	0.104	0.92 (0.67, 1.26)	0.610
2009+	1.51 (1.22, 1.86)	<.001	1.72 (1.16, 2.56)	0.007
***Hepatitis B/C coinfection, n(%)***				
Negative	1.00		1.00	
Positive	0.77 (0.63, 0.93)	0.008	1.01 (0.67, 1.53)	0.948
Not tested	0.73 (0.57, 0.94)	0.013	0.44 (0.27, 0.72)	0.001
***CD4 count***				
per 100 cells/mmc lower	1.22 (1.15, 1.29)	<.001	1.22 (1.11, 1.34)	<.001
***Viral load***				
per log10 copies/mL higher	1.37 (1.20, 1.57)	<.001	1.06 (0.90, 1.25)	0.501
***CD8 count***				
per 100 cells/mmc higher	0.96 (0.94, 0.98)	<.001		
***ADE group, n(%)***				
Group A	1.00		1.00	
Group B	2.18 (1.63, 2.90)	<.001	1.63 (1.05, 2.53)	0.028
Group C	2.04 (1.64, 2.55)	<.001	1.01 (0.71, 1.42)	0.962
***Haemglobin***				
per 100 g/dL higher	0.63 (0.37, 1.07)	0.089		
***White Blood Cell***				
per 1000 cells/mmc higher	0.91 (0.86, 0.95)	<.001		
***ALT***				
per 100 IU/L higher	1.07 (0.86, 1.34)	0.524		
***AST***				
per 100 IU/L higher	1.06 (0.84, 1.34)	0.627		
***eGFR***				
per 50 100 mls/min/1.73 m^2 higher^	1.29 (0.91, 1.82)	0.147		
***Reason for enrolment, n(%)***				
No indications for ART	1.00		1.00	
Patients decision	1.12 (0.77, 1.62)	0.564	0.63 (0.37, 1.09)	0.097
Contraindications	0.64 (0.23, 1.78)	0.396	0.19 (0.03, 1.43)	0.107
Recent HIV diagnosis	2.04 (1.51, 2.76)	<.001	1.86 (1.17, 2.93)	0.008
Recent access to care	1.45 (1.01, 2.08)	0.045	1.26 (0.77, 2.08)	0.361
***No. of diagnoses, n(%)***				
Per 1 additional	1.21 (1.02, 1.44)	0.033	1.05 (0.79, 1.40)	0.723

#### Description of people not starting ART

Two hundred and two patients were still off-ART. Of these 8 had died and the remaining 194 had not commenced ART at the time that they were last seen alive (Figure 1). The reasons for not starting ART or being censored are not collected in our database. However, for the 633 patients who were diagnosed before or at the time of enrolment we have the reason for being ART-naïve. The main reasons were: recent seroconversion (68%), recent access to care (14%) and patients' reasons (10%). Overall, the distribution of reasons for being ART-naive at enrolment was similar in people who subsequently started ART or those who remained off-ART (data not shown).

#### Assessment of missing data

Some of the variables in the model were fitted as categorical and individuals with missing data for these factors have been grouped separately as ‘value unknown’. People with missing data for variables included as continuous (e.g. CD4 count at the date of AIDS diagnosis) were, however, excluded from the multivariable calculations. People included and excluded (due to missing values of these variables) were statistically different for some demographic characteristics but large differences were not observed (data not shown) with the exception of year of diagnosis (more recent year of AIDS diagnosis in excluded patients [2007 on average], compared to patients included in multivariable model [2000]). People who were diagnosed with HIV in more recent years were more likely to have missing data for CD4 count or viral load at time of their AIDS diagnosis. This could be due to the fact that data collection was modified approximately in 2005 to collect only one historical value of these markers at entry in the cohort (two values were instead recorded in previous years).

## Discussion

The results of our analysis show that in recent years Italian clinicians tend to initiate ART more promptly than in the past after a diagnosis of AIDS. Nevertheless, even in recent years the overall probability of starting ART seems to remain low (43% by 30 days from the AIDS diagnosis) with a median time to ART initiation of 21 days. Indeed, these estimates are considerably lower than what has been suggested as optimal time of starting ART on the basis of the results of the ACTG 5164 trial [4]. Even in the subset of people who were diagnosed under prospective follow-up in the cohort, the estimate remains low (38% instead of 33% by 30 days). Of note, however, our estimates refer to any diagnosis of OI while in the trial 75% of patients had been diagnosed with Pcp and cases of TB were excluded.

To our knowledge ours is the first analysis evaluating whether there has been a change in the timing of ART initiation in clinical practice over time in cART-naive patients presenting for care with different AIDS-defining conditions.

Recently, Geng et al reported a 3-fold increase in the rate of ART initiation and an increase in the probability of early ART from 7% to 50% after the diffusion of ACTG 5164 findings in a single clinical center in the USA uniquely in patients diagnosed with Pcp [9].

In a retrospective European and Canadian multi-cohort study, aimed to analyse the clinical progression of AIDS-presenter patients, the proportion of patients starting ART by 30 days from diagnosis was 50% when considered the overall study population, ranging from 70% in people diagnosed with KS to 30% in those with NHL, with wide variation in the time to initiation also according to country of origin [2]. Of note, not all individuals studied by us were AIDS presenters, 462/720 (64%) developed AIDS after enrolment in the cohort.

There are a number of potential concerns and obstacles to the early initiation of ART in patients presenting with opportunistic conditions: the risk of immune reconstitution inflammatory syndrome, the concern for poor adherence to multi-drug treatment, for drug-drug interactions potentially leading to adverse events or poor treatment responses, and the difficulties in taking or tolerating oral medications for critically ill patients. All these represent potential reasons for delaying antiretroviral treatment and may partially explain our findings [17]. Indeed, people with AIDS defining conditions for which ART represents the only treatment option were treated earlier, whereas those whose conditions were treatable with medications having potential drug-drug interaction with antiretrovirals were treated later.

Although both current Italian and International guidelines report a general agreement on the net benefit of a prompt start of ART in people with AIDS, data evaluating the effect of early ART on specific opportunistic conditions other than Pcp, TB and cryptococcosis are very limited, and this could lead the clinicians to hesitate in ART initiation in people diagnosed with less well-studied conditions such as CMV disease or esophageal candidiasis or toxoplasmosis.

In our study population, TB accounted for 14% of people with AIDS. When we looked at people diagnosed with TB alone the median time to starting ART was 142 days (95% CI: 97–190). Of note, we found that the effect of calendar period was stronger in group A (including TB) than in group C (including Pcp); this was confirmed even after restricting the analysis to people with TB or Pcp only (data not shown). This may represent an unexpected result, because recommendation on earlier start of ART based on the data of ACTG 5164 concerned more specifically Pcp rather than TB. It can be speculated that other factors may have affected a prompt initiation of ART in people with TB such as a more extensive use of NNRTI in the recent years (with less concerns in terms of drug-drug interactions), or a progressive increase of awareness of findings of randomized clinical trials on TB [18]–[21]. In addition, immune reconstitution inflammatory syndrome (IRIS) is often observed after starting treatment for specific disorders (i.e., TB, CMV, criptococcosis) and this might be another reason why clinicians are less likely to promptly start ART in people with TB.

The opposite trend was seen for people diagnosed with cryptococcal meningitis (less chance to initiate ART in recent periods) this might be due to increasing evidence for a worst survival in patients with this pathology starting ART earlier [21]–[22].

Investigating the predictors of time to ART initiation, we found that patients whose reason for enrolment in Icona was a recent diagnosis of HIV infection, were more likely to be promptly started on ART compared to patients who had no indication for initiating ART. These are likely to be people who were diagnosed with HIV when they presented with AIDS and it is conceivable that their time to ART initiation is the shortest. Of note indication for starting ART have also been modified in guidelines over time but to a lower extent for people with already a diagnosis of AIDS.

Moreover, patients of non-Italian nationality tended to start ART later than Italian patients. Some concerns regarding the retention in care of migrant patients might explain the conservative approach of clinicians in starting ART in this subset of patients.

Our analysis has several limitations.

First, although a severity criterion could be establish for the classification of ADE, for a given pathology, we do not collect data on the degree of severity or progression of the disease. For example, it could be hypothesized that in case of greater severity, requiring also more intensive care, ART was not started immediately because patients were not able to take it. Moreover, AIDS diagnoses are typically made using a presumptive diagnostic criterion according to CDC guidelines. As a consequence people may be started on ART after a presumptive and before a confirmative diagnosis and excluded from the analysis as a result. This may have led to selection bias as well as over-estimation of the estimated time to start ART. However, only 88 of the 720 diagnoses (12%) analyzed here were made using a presumptive diagnostic criterion.

Furthermore, we did not perform a survival analysis with endpoint death so we do not know what the impact of such a conservative approach may be on mortality in a real practice setting. We are indeed planning to investigate the association between the strategies of starting ART within 15 days vs. starting within 30 days vs delaying for longer than 30 days or not start at all and the risk of death using g-computation in a separate report [23]. Patients with missing data have been excluded from the multivariable Cox regression analysis. However, included and excluded patients were similar for the other measured characteristics and therefore selection bias is unlikely. There was a large difference in calendar year of diagnosis likely to be due to a change in modality of data collection over time which is unrelated to time to ART initiation.

In conclusion, in a real life setting of patients seen for care in Italy, we show that the time from AIDS diagnosis to ART initiation was significantly shorter in more recent years, even though still considerably longer than what has been reported as beneficial on survival in AIDS patients. Reasons for that could be a delay in the implementation of guidelines in clinical practice, but could also be due to real difficulties in treating these patients concomitantly for opportunistic conditions and for HIV itself. Similar prospective studies with data collection enrichment are warranted to explore in details the specific barriers to ART initiation in patients presenting with ADE.

## Supporting Information

Figure S1 **Kaplan-Meier estimates of the cumulative proportion of patients starting ART by 30 days from ADE diagnosis, according to different combination of period of starting ART and group of ADEs.**
(TIFF)

Table S1 **Characteristics of patients according to different groups of ADEs.**
(DOCX)
