# Supplementary figures and images for: Timing of Antiretroviral Therapy Initiation after a First AIDS-Defining Event: Temporal Changes in Clinical Attitudes in the ICONA Cohort
Source: PLoS One. 2014 Feb 27;9(2):e89861. doi: 10.1371/journal.pone.0089861 (PMC3937396; doi:10.1371/journal.pone.0089861)

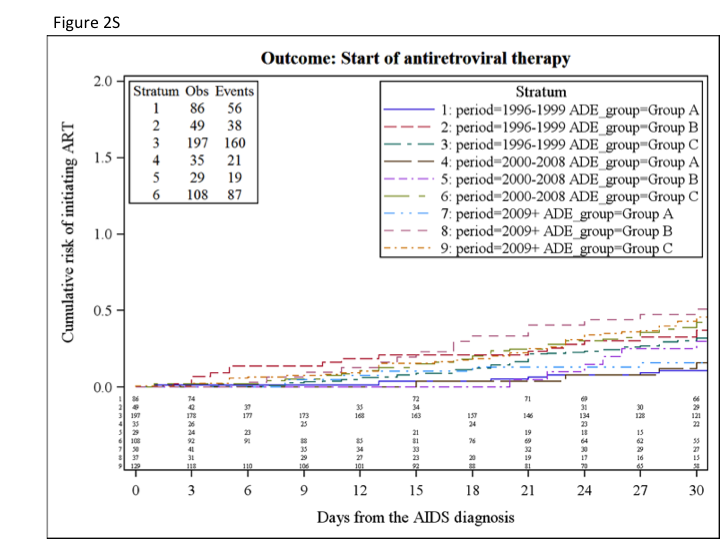

Supplement: Figure S1 — Kaplan-Meier estimates of the cumulative proportion of patients starting ART by 30 days from ADE diagnosis, according to different combination of period of starting ART and group of ADEs. (TIFF) [file pone.0089861.s001.tiff]
