# Supplementary material for: Timing of Antiretroviral Therapy Initiation after a First AIDS-Defining Event: Temporal Changes in Clinical Attitudes in the ICONA Cohort
Source: PLoS One. 2014 Feb 27;9(2):e89861. doi: 10.1371/journal.pone.0089861 (PMC3937396; doi:10.1371/journal.pone.0089861)
Supplement: Table S1 — Characteristics of patients according to different groups of ADEs. (DOCX) [file pone.0089861.s002.docx]

Supplemental Material.

Table S1 Characteristics of patients according to different groups of ADEs

| **Characteristics at AIDS diagnosis** | **Group A** | **Group B** | **Group C** | **p-value^*^** |
| --- | --- | --- | --- | --- |
|  | N= 171 | N= 115 | N= 434 |  |
| ***Age, years*** |  |  |  |  |
| Median (IQR) | 38 (33, 45) | 40 (36, 48) | 36 (31, 41) | <.001 |
| ***Gender, n(%)*** |  |  |  | 0.038 |
| Female | 129 (29.7%) | 21 (18.3%) | 52 (30.4%) |  |
| ***Nation of birth, n(%)*** |  |  |  | <.001 |
| Italian | 372 (85.7%) | 100 (87.0%) | 104 (60.8%) |  |
| ***Mode of HIV Transmission, n(%)*** |  |  |  | 0.064 |
| IDU | 102 (23.5%) | 21 (18.3%) | 44 (25.7%) |  |
| Homosexual contacts | 95 (21.9%) | 38 (33.0%) | 29 (17.0%) |  |
| Heterosexual contacts | 200 (46.1%) | 46 (40.0%) | 78 (45.6%) |  |
| Other/Unknown | 37 (8.5%) | 10 (8.7%) | 20 (11.7%) |  |
| ***Calendar year of AIDS diagnosis*** |  |  |  |  |
| Median (range) | 2002 (1996, 2013) | 2004 (1997, 2013) | 2000 (1997, 2013) | 0.494 |
| ***HBsAg, n(%)*** |  |  |  | 0.466 |
| Negative | 349 (80.4%) | 100 (87.0%) | 135 (78.9%) |  |
| Positive | 7 (1.6%) | 1 (0.9%) | 4 (2.3%) |  |
| Not tested | 78 (18.0%) | 14 (12.2%) | 32 (18.7%) |  |
| ***HCVAb, n(%)*** |  |  |  | 0.470 |
| Negative | 244 (56.2%) | 72 (62.6%) | 96 (56.1%) |  |
| Positive | 114 (26.3%) | 30 (26.1%) | 50 (29.2%) |  |
| Not tested | 76 (17.5%) | 13 (11.3%) | 25 (14.6%) |  |
| ***CD4 count, cells/mmc*** |  |  |  |  |
| Median (IQR) | 35 (17, 91) | 69 (32, 230) | 156 (59, 397) | <.001 |
| ***Viral load, log10 copies/mL*** |  |  |  |  |
| Median (IQR) | 5.32 (4.81, 5.70) | 5.46 (4.88, 5.88) | 4.91 (4.11, 5.58) | <.001 |
| ***CD8 count, cells/mmc*** |  |  |  |  |
| Median (IQR) | 455 (234, 744) | 557 (305, 952) | 739 (429, 1241) | <.001 |
| ***CD4/CD8 ratio, cells/mmc*** |  |  |  |  |
| Median (IQR) | 0.10 (0.04, 0.18) | 0.14 (0.07, 0.22) | 0.27 (0.12, 0.49) | <.001 |
| ***ADE group, n(%)*** |  |  |  | 0.021 |
| Group A | 0 (0.0%) | 0 (0.0%) | 171 (100.0%) |  |
| Group B | 0 (0.0%) | 115 (100.0%) | 0 (0.0%) |  |
| Group C | 434 (100.0%) | 0 (0.0%) | 0 (0.0%) |  |
| ***Haemglobin, g/dL*** |  |  |  |  |
| Median (IQR) | 12 (10, 13) | 12 (10, 13) | 11 (10, 13) | 0.634 |
| ***White Blood Cell, cells/mmc*** |  |  |  |  |
| Median (IQR) | 4200 (3000, 6100) | 4400 (3130, 5980) | 4910 (3300, 7110) | 0.080 |
| ***ALT, IU/L*** |  |  |  |  |
| Median (IQR) | 32 (21, 51) | 30 (18, 46) | 34 (23, 68) | 0.137 |
| ***AST, IU/L*** |  |  |  |  |
| Median (IQR) | 35 (24, 55) | 29 (21, 43) | 37 (24, 62) | 0.034 |
| ***eGFR, 100mls/min/1.73m^2^*** |  |  |  |  |
| Median (IQR) | 107.7 (86.94, 126.4) | 107.9 (89.54, 130.8) | 98.68 (86.68, 114.3) | 0.159 |
| ***Reason for enrolment, n(%)*** |  |  |  |  |
| No indications for ART | 20 (4.7%) | 10 (8.8%) | 19 (11.3%) |  |
| Patients decision | 41 (9.6%) | 12 (10.5%) | 29 (17.3%) |  |
| Contraindications | 4 (0.9%) | 1 (0.9%) | 2 (1.2%) |  |
| Recent HIV diagnosis | 287 (67.1%) | 79 (69.3%) | 87 (51.8%) |  |
| Recent access to care | 64 (15.0%) | 9 (7.9%) | 25 (14.9%) |  |
| Physician decision | 9 (2.1%) | 2 (1.8%) | 5 (3.0%) |  |
| Unknown | 3 (0.7%) | 1 (0.9%) | 1 (0.6%) |  |
| ***Time from enrolment to ADE, days*** |  |  |  |  |
| Median (range) | 0 (0, 3685) | 0 (0, 3333) | 0 (0, 4358) | <.001 |
| ***Calendar year of HIV diagnosis*** |  |  |  |  |
| Median (IQR) | 2000 (1997, 2009) | 2001 (1997, 2009) | 1999 (1994, 2007) | 0.034 |
| ^*^Chi-square or Wilcoxon test as appropriate | | | | |

Figure S1

Kaplan-Meier estimates of the cumulative proportion of patients starting ART by 30 days from ADE diagnosis, according to different combination of period of starting ART and group of ADEs.
